# Aquaporin-4 and Parkinson’s Disease

**DOI:** 10.3390/ijms25031672

**Published:** 2024-01-30

**Authors:** Ksenia V. Lapshina, Irina V. Ekimova

**Affiliations:** Laboratory of Comparative Thermophysiology, Sechenov Institute of Evolutionary Physiology and Biochemistry of RAS, 194223 Saint Petersburg, Russia; irina-ekimova@mail.ru

**Keywords:** aquaporin-4, α-synuclein, Parkinson’s disease, neurodegeneration, glymphatic system, pathophysiology, drug target

## Abstract

The water-selective channel aquaporin-4 (AQP4) is implicated in water homeostasis and the functioning of the glymphatic system, which eliminates various metabolites from the brain tissue, including amyloidogenic proteins. Misfolding of the α-synuclein protein and its post-translational modifications play a crucial role in the development of Parkinson’s disease (PD) and other synucleopathies, leading to the formation of cytotoxic oligomers and aggregates that cause neurodegeneration. Human and animal studies have shown an interconnection between AQP4 dysfunction and α-synuclein accumulation; however, the specific role of AQP4 in these mechanisms remains unclear. This review summarizes the current knowledge on the role of AQP4 dysfunction in the progression of α-synuclein pathology, considering the possible effects of AQP4 dysregulation on brain molecular mechanisms that can impact α-synuclein modification, accumulation and aggregation. It also highlights future directions that can help study the role of AQP4 in the functioning of the protective mechanisms of the brain during the development of PD and other neurodegenerative diseases.

## 1. Introduction

Parkinson’s disease (PD) is a progressive, chronic, and multisystem neurodegenerative disease that is the second most common neurodegenerative disorder after Alzheimer’s disease (AD). PD predominantly affects the elderly, and owing to increasing life expectancy, the number of patients is steadily increasing [1,2]. A key characteristic feature of PD is the extensive loss of dopamine (DA)-ergic neurons of the substantia nigra pars compacta (SNpc), which leads to a decrease in DA level in the striatum and the development of a complex of motor symptoms, such as tremor, rigidity, postural instability, and bradykinesia [3,4,5]. In addition to the development of nigrostriatal neurodegeneration, PD affects many other brain areas, such as the olfactory structures, locus coeruleus, hypothalamus, and other brain regions [6,7,8]. The involvement of these structures can lead to the development of different non-motor symptoms such as sleep disturbances, cognitive impairment, depression, anosmia, and constipation [9]. Moreover, according to the “dual-hit theory,” the pathology of PD extends well beyond the central nervous system (CNS) because Lewy bodies (LBs) have been detected in the myenteric plexus [10,11]. Thus, that makes PD not only a “brain disease” but a multiorgan disease [12]. A feature of this disease is its long-term development (≥10 years) without the manifestation of clinically significant motor dysfunction, which leads to late diagnosis [13]. Despite all efforts undertaken to design therapeutic approaches aimed at PD treatment, this area does not lose its relevance since PD remains an incurable disease. Existing treatments are only symptomatic, and clinically approved therapeutic approaches that effectively protect brain cells from pathological processes and can slow or stop neurodegeneration are not yet available. Progress in the treatment of PD has been associated with the development of early diagnostic and pathogenetically significant neuroprotective strategies. Further studies on the pathogenesis of this disease are required to address this problem.

The accumulation of pathological forms of α-synuclein protein is considered a molecular basis for the development of neurodegeneration in PD [14,15,16,17]. This protein is prone to misfolding and post-translational modifications, which result in the formation of cytotoxic oligomers, fibrils and aggregates that accumulate in neurons of the SNpc and extranigral brain areas, as well as in intercellular spaces. Oxidative stress, mitochondrial dysfunction, impaired protein degradation, the post-translational modifications of α-synuclein, inflammation, defects in chaperone-mediated conformational control, increased calcium level and other deleterious events in neurodegenerative diseases are thought to be key factors evoking α-synuclein aggregation [17,18,19,20,21,22]. Moreover, α-synuclein can be secreted into the intercellular space and spread in a prion-like manner [14,16,23]. This multifaceted damaging effect of α-synuclein is one of the reasons why it is difficult to develop an effective therapeutic approach. Breakthrough studies of these mechanisms are essential to search for new approaches aimed at preventing α-synuclein spreading and lowering its toxicity.

One of the important achievements of the last decade was the development of ideas about the glymphatic system that clears the brain parenchyma from various metabolites (including amyloidogenic proteins), as well as an active investigation of the role of meningeal lymphatic vessels and deep cervical lymph nodes in eliminating metabolites [24,25,26,27]. Water channel aquaporin-4 (AQP4), which provides bidirectional water exchange, is an important component of the glymphatic system [24]. Therefore, studies on the relationship between the quality of glymphatic system function and the pathogenesis of neurodegenerative diseases have attracted much attention from researchers. However, the main focus has been on investigating the impact of AQP4 dysfunction on amyloid-β and AD development [28,29,30,31,32]. There are very few data on the interaction between AQP4 and PD development, especially the formation of α-synuclein pathology complicated by both extra- and intracellular distribution of synuclein [33,34].

Recently, it was shown that AQP4 deficiency in mice leads to the aggravation of α-synuclein pathology in PD animal models [35,36]. These data suggest that this channel is involved in PD pathogenesis. In AD animal models, AQP4 dysfunction leads to amyloid-β and tau accumulation, but the mechanisms underlying this accumulation are controversial [37,38,39,40].

Thus, despite the first evidence that AQP4 dysfunction can play an important role in the mechanisms of α-synuclein accumulation, the underlying mechanisms remain poorly understood. Current studies have focused mainly on the processes of glymphatic/lymphatic clearance of amyloidogenic proteins, whereas AQP4 dysfunction may critically affect brain water homeostasis and microenvironment as well as cellular functions, which are also involved in waste clearance and brain well-being control [41,42]. However, this line of research appears to have been underestimated. Our review aimed to analyze and summarize the current data on the relationship between AQP4 dysfunction and α-synuclein accumulation, consider the possible effects of AQP4 dysregulation on brain cellular mechanisms that can affect α-synuclein pathology, and explore possible future directions and their relevance for translational research.

## 2. α-Synuclein and Its Role in PD Development

α-Synuclein, encoded by the SNCA gene, is a 14 kDa protein that is located mostly in presynaptic terminals, neuronal cytosol and nucleus [18,43,44]. It is involved in the regulation of the vesicular pool and the trafficking and transmission of nerve impulses [18]. However, α-synuclein can affect mitochondrial and endoplasmic reticulum (ER) homeostasis, lysosome/phagosome function, fatty acid binding, physiological regulation of certain enzymes, and cytoskeleton organization [44]. Some studies have suggested that α-synuclein knockout can be harmful, causing neuronal cell death and altered neurotransmitter responses, whereas other studies have shown that it can be partly protective against DA depletion [45,46,47,48]. Genetic inactivation of α-synuclein in mice affects embryonic development of DA-ergic neurons in the SN, leading to its loss. No such changes were observed in the ventral tegmental area [49]. Loss of synuclein family members, not just a specific one, causes widespread changes in EEG spectral profiles in mice, suggesting that an imbalance of synucleins is responsible for these changes [50]. Thus, evidence from the literature suggests that α-synuclein may play an important role in the vitality of brain cells and that its deficiency may have deleterious effects. However, the physiological function of α-synuclein in cells is not fully understood.

Being natively unfolded, α-synuclein can be classified as an intrinsically disordered protein (IDP) class [51]. However, they are prone to misfolding and modification. Owing to its low hydrophobicity and high net charge, environmental conditions can affect the properties of α-synuclein [52]. Some A53T and A30P mutations and gene triplication can genetically determine the increased level of α-synuclein and predispose to PD development [53]. α-Synuclein monomers can aggregate into oligomers, protofibrils, and fibrils [18,51]. Eventually, α-synuclein becomes one of the main components of amyloid inclusions known as LBs and Lewy neurites, which are hallmarks of PD, multiple system atrophy (MSA) and dementia with LB (DLB) [53,54,55]. LBs formation is supposed to be associated with synaptic dysfunction and abnormal organization of the neuronal cytoskeleton. Their composition may vary and influence degeneration in PD [56,57]. Another hypothesis is that the formation of LBs might be a neuroprotective mechanism aimed at decreasing neurotoxicity in affected neurons [58,59].

α-Synuclein oligomers are characterized by a wide variety of structures. However, certain types of α-synuclein oligomers have yet to be distinguished [60]. They are formed depending on the specified conditions and are characterized by different targets, actions, and capabilities to aggregate. Another feature of this protein is the presence of sites for post-translational modifications, such as phosphorylation (especially Ser129), acetylation, nitration, SUMOylation, and ubiquitination [19,20,61,62,63,64]. These post-translational modifications can occur due to different factors, both endogenous and exogenous. Almost all of these facilitate oligomerization and aggregation of α-synuclein, contributing to the development of α-synuclein pathology. However, some post-translational modifications, such as phosphorylation of Tyr125 and Tyr39, as well as acetylation, can reduce oligomerization and aggregation [65,66,67]. Recently, it was reported that neuronal activity may occur as a physiological regulator of α-synuclein phosphorylation at Ser129 [68]. This form is considered to be one of the most neurotoxic and prone to oligomerization and aggregation; however, data suggest that the properties of α-synuclein phosphorylated at Ser129 remain incompletely studied.

According to modern data, α-synuclein can be found throughout the CNS, enteric nervous system, sympathetic ganglia, submandibular glands, skin, and other areas [18,69]. It is known that α-synuclein can be secreted into the extracellular space and taken up by neighboring cells via receptor-mediated endocytosis and phagocytosis [70,71]. α-Synuclein can behave in a prion-like manner, causing the alteration of physiological α-synuclein in other cells and contributing to the progression of this pathological process [14,16]. The development of α-synuclein pathology in PD can be genetically determined by duplication and triplication [53,72]. In idiopathic PD, as well as in MSA and DLB, α-synuclein is affected by different triggers, such as aging, oxidative stress, nitrate, decreased activity of chaperones and protein degradation systems, DA dysregulation, and accumulation of other amyloidogenic proteins [18]. These factors contribute to the development of α-synuclein pathology. However, we believe that this list is incomplete. Furthermore, mutations in some genes may predispose individuals to the development of α-synuclein pathology and exaggerate the impact of these triggers [73]. Pathological α-synuclein possesses multifaceted deleterious actions, disturbing synaptic function and axonal transport, penetrating membrane branes, forming pore-like channels, disrupting membrane lipid bilayers, evoking mitochondrial damage, neuroinflammation, ER stress, dysfunction of lysosomal autophagy, and UPS systems aimed at the degradation of aberrant proteins [18,22,74]. Such broad-spectrum impacts of pathological α-synuclein underlie the degeneration of DA-ergic neurons of the SNpc and other brain structures, which leads to the development of a complex of PD-associated motor and non-motor symptoms [6,9].

In this regard, the search for pharmacological drugs or other approaches aimed at reducing cytotoxicity and preventing the accumulation and distribution of α-synuclein appears to be an important and relevant direction. However, it faces many challenges, such as different primary causes of pathological α-synuclein accumulation (genetic or idiopathic), the impact of peripherally originated α-synuclein, the multifaceted cytotoxic action of α-synuclein, the diversity of oligomeric forms, the extent to which neurons and astrocytes are involved in the development of α-synuclein pathology, predisposing somatic and/or neurological co-pathologies, and aging [75].

Currently, efforts are being made to design different methods to affect α-synuclein pathology for the treatment of PD. Inhibitors aim to prevent α-synuclein internalization and strain-specific antibodies that decrease the levels of α-synuclein in the extracellular space [76]. Another promising approach to identifying specific inhibitors of α-synuclein toxic species is the use of aptamers that bind with high specificity to different truncated forms of α-synuclein fibrils with no cross-reactivity toward other amyloid fibrils [77]. These aptamers can be used as tools in research, diagnostics, and therapy. Some strategies are aimed at improving proteostasis, such as small molecule compounds aimed at preventing α-synuclein misfolding or chaperone induction and aggregate resorption [78,79,80,81,82] or PROTAC (Proteolysis-Targeting Chimera) [83], which provides chimera chemicals inducing selective degradation of α-synuclein aggregates in vitro and in vivo, and autophagy activator KYP-2047 [84]. Despite extensive investigations aimed at designing therapy to treat PD, there are currently no clinically approved approaches to eliminate α-synuclein toxicity or prevent the spread of pathological α-synuclein. Taken together, further investigation of the molecular mechanisms underlying the development of α-synuclein pathology and PD pathogenesis (as well as other synucleinopathies) is necessary to identify new targets and design new treatment approaches.

Increased knowledge of the brain glymphatic system, which provides clearance of brain tissue, suggests that this pathway may be a novel approach to developing a therapeutic approach for PD. The water channel AQP4, which provides the functioning of the glymphatic system, appears to be a promising target for modulation.

## 3. Aquaporin-4 and Its Role in PD Development

### 3.1. AQP4: A Brief Overview

Aquaporins, also known as water channels, are membrane proteins that enable the selective transport of water and small molecules into and out of cells [85]. The family of aquaporins found in mammals consists of 13 members, namely AQP0 to AQP12 [85]. The first member of this family was uncovered in 1986 and was characterized in 1992, being subsequently referred to as aquaporin 1 (AQP1) [86,87]. AQP4 was originally cloned in 1994 from rat lung and brain, as well as human brain [88,89,90]. AQP4 is the most abundant form of aquaporin in the CNS and is expressed widely throughout the brain, particularly at the brain-blood interfaces in the brain and spinal cord [41,91]. AQP4 is localized on the plasma membrane of astrocytes and ependymal cells but is most abundantly expressed in astrocytic endfeet surrounding the blood vessels [41,42,91,92]. This significantly polarized expression of AQP4 plays a crucial role in water transport and the maintenance of cerebral water balance [41,42,91,92]. It has been found that AQP4 can also be present in the kidneys, retina, heart, skeletal muscle, and other organs, as well as in tumors [41,89,90,91,92].

AQP4 consists of monomers weighing approximately 30 kDa, with six transmembrane alpha-helical domains [92,93]. The monomers combine into tetramers, but each of them functions as a separate channel [93]. AQP4 can be arranged in supramolecular structures called orthogonal arrays of particles (OAPs) [93,94,95]. AQP4 is anchored to the membrane via a cytoskeletal dystrophin–glycoprotein complex (DGC), which includes α-syntrophin, dystroglycan (DG), utrophin, α-dystrobrevin, and dystrophin Dp71 [96,97]. This complex also regulates AQP4 membrane localization [97].

There are two main AQP4 isoforms: M23 and M1 [98,99,100]. An alternative translational read-through mechanism provides the possibility of producing two extended AQP4 isoforms, M23ex and M1ex, in humans, rats, and mice [100,101,102]. These isoforms are thought to be modulators of function and assembly of OAPs [100]. Moreover, phosphorylation at specific sites may transiently regulate channel gating and water permeability [102]. In primary cultures of rat astrocytes, a large proportion of M23 protein is derived from M1 mRNA translation, and the M1/M23 ratio can be modulated by a multiple-site leaky scanning mechanism and reinitiation mechanism. This is a way to modulate the M1/M23 ratio and, consequently, the formation of OAPs [100]. These mechanisms are likely to be shared by different species, including humans, and can also play a role in pathophysiological conditions. AQP4 is an important regulator of water homeostasis in the brain and may act as an osmoreceptor [41,89]. In addition, it was found that this form of aquaporin plays an important role in astrocyte migration and regulates nerve signal transmission [41,103,104].

However, the main focus has been on AQP4 after its involvement in the glymphatic system was identified. In 2012, a group of researchers led by M. Nedergaard made an important assumption, showing that cerebrospinal fluid (CSF) can flow through the para-arterial spaces into the brain parenchyma, mix with interstitial fluid containing various waste products of brain cells, and then through para-venous spaces, direct fluid with metabolites to the meningeal and cervical lymphatic vessels and nodes to ensure the clearance of the brain parenchyma [24,25,26,27,105,106]. This pathway has been termed the “glymphatic system” and is thought to function most efficiently during sleep [105,106]. After this discovery, studies on the relationship between the quality of the glymphatic system/lymphatic system functioning and the pathogenesis of neurodegenerative diseases have attracted much attention from researchers.

The expression of AQP4 has been found to vary in different brain structures. In mice, the least pronounced expression was found in the cerebral cortex and striatum, with a higher expression in the cerebellum, olfactory bulb, brainstem and the spinal cord [107]. In another study, the authors compared the levels of AQP4 protein in different brain regions (forebrain, subcortical areas, and brainstem) and found the highest level in the cerebellum and lower expression in the cortex and hippocampus [108]. Interestingly, AQP4 immunoreactivity differed between pars reticulata and pars compacta; the intensity of AQP4 staining in pars compacta was lower than that in pars reticulata [108]. This can be a result of the greater representation of astroglia in pars reticulata than in pars compacta [108]. In another study, when comparing the expression of AQP4 in the cerebral cortex and SN, it was found that the expression of AQP4 is much higher in the SN, both in the perivascular processes of astrocytes and in thinner processes in the neuropil [109]. Such heterogeneity in expression level may indicate different glymphatic clearance intensities in different structures. These data suggest that region-specific AQP4 expression may reflect local peculiarities in water and ion homeostasis, synaptic plasticity, and waste clearance mechanisms.

Aging is known to be a risk factor for the development of PD and other neurodegenerative diseases [110], and it has been suggested to be one of the most important factors affecting AQP4 localization and expression as well. A post-mortem study found that the accumulation of amyloid-β was associated with impaired perivascular localization of AQP4, suggesting that this may be a contributing factor to impaired clearance and make the aging brain much more vulnerable to the accumulation of toxic proteins and oligomers, which induce neurodegeneration [30]. However, a more recent study revealed the loss of perivascular AQP4 localization in the post-mortem frontal cortical gray matter of subjects with AD compared to cognitively intact subjects, which was accompanied by amyloid-β accumulation and cognitive decline before the onset of dementia [111]. These data suggest that loss of perivascular AQP4 may contribute to AD development and progression [111]. In mice, aging leads to a dramatic decline in the efficiency of exchange between the subarachnoid CSF and the brain parenchyma, which is accompanied by the impairment of clearance of labeled amyloid β from the brain parenchyma, reduction in the vessel wall pulsatility of intracortical arterioles, and loss of perivascular AQP4 polarization along the penetrating arteries [112]. Another study has demonstrated that WT and TgSwD animals (developing age-related amyloid accumulation) were characterized by an impairment of perivascular localization of AQP4 in aged (24 months) compared to 6 months old. Moreover, TgSwD mice were characterized by amyloid accumulation and development of gliosis [113]. A post-mortem human study demonstrated that aging is associated with increased AQP4 expression in cortices [30,114]. Similar changes in AQP4 expression were found in mice [115,116]. Taken together with the aforementioned data concerning the age-related loss of perivascular AQP4 polarization, it can be supposed that the increased expression of AQP4 in aged brains can be a physiological reaction compensating for AQP4 dysfunction. Thus, these studies suggest that there is a close interconnection between AQP4-mediated glymphatic waste clearance, aging and neurodegenerative processes, which underlie PD, AD and other neurodegenerative diseases [117].

### 3.2. AQP4 Dysregulation in PD

Data available in the literature suggest a relationship between the functional activity of AQP4 and the accumulation of amyloidogenic proteins [24,25]. The extent to which the glymphatic system influences the clearance of pathological forms of α-synuclein in the human brain and the progression of PD-like features remains poorly understood. Specifically, it has been shown that PD is accompanied by a decrease in AQP4 mRNA levels in blood serum, indicating lowered expression [118]. It was later shown that there was a negative correlation between α-synuclein and AQP4 levels in the neocortex [28]. The authors suggested that AQP4-expressing reactive astrocytes might affect the accumulation of α-synuclein in the neocortex of patients with PD. This indicated a link between AQP4 and the development of α-synuclein pathology in PD. Some studies have indirectly indicated the involvement of the water channel AQP4 in the pathogenesis of PD [34,119,120,121,122]. In patients with PD, magnetic resonance imaging (MRI) revealed an accumulation of free water in the SN tissue, which increased with the progression of PD. According to the author’s observations, it can predict upcoming changes in motor and cognitive functions [34]. Later, it was demonstrated that other neurodegenerative disturbances (MSA and progressive supranuclear palsy), which are also associated with nigral degeneration, are characterized by the accumulation of free water in this brain region [119]. These data may indicate dysregulation of water homeostasis in this brain region; however, the underlying mechanism remains unknown. Recent studies have shown that some variants of the Aqp4 gene single-nucleotide polymorphism (SNP) are associated with a higher risk of PD development, the appearance of cognitive impairments, PD-associated regional brain activity, and clinical phenotypes [120,121,122]. It is noteworthy that Y. Fang and coauthors revealed the existence of the Aqp4 SNP form, which was associated with the faster progression of cognitive impairment and amyloid-β in CSF of PD patients [120]. Recently, the signs of glymphatic dysfunction have been revealed not only in the clinical stage of PD but also in the prodromal stage in patients with idiopathic rapid eye movement sleep behavior disorder and PD [123]. Consequently, enhancing the efficiency of the glymphatic system in the early stages of PD may be a potential strategy to intervene in the pathogenic processes. However, the accumulated clinical data in PD patients are scarce and do not allow us to provide a detailed characterization of the function of the glymphatic system in the pathophysiology of PD and to identify the main molecular targets for enhancing the function of the glymphatic system and eliminating the dysfunction of water channel AQP4.

Studies performed in animal models of PD support clinical evidence for the role of AQP4-mediated glymphatic system dysfunction in the pathogenesis of PD [124]. It was found that PD models can induce alterations in AQP4. In mice overexpressing A53T-α-synuclein, the expression/polarization of AQP4 in the SN was reduced, and glymphatic activity was suppressed [36]. Interestingly, in another model of PD induced by 1-methyl-4-phenyl-1,2,3,6-tetrahydropyridine (MPTP), there was no decrease but rather an increase in the expression of AQP4 in the SN in mice [109].

The accumulation of α-synuclein in the brain parenchyma during AQP4 dysfunction has also been indicated in studies performed using Aqp4 knockout mice. Such animals, in which human A53T α-synuclein was overexpressed in the SNpc, exhibited increased accumulation of α-synuclein monomers 2 weeks after the start of the experiment, which, according to the authors, indicated a close interaction between AQP4-mediated by the glymphatic system and α-synuclein located in the brain parenchyma [36]. When α-synuclein fibrils were injected into the dorsal striatum of Aqp4 knockout mice, after three months, there was an increased accumulation of α-synuclein phosphorylated at Ser129 as part of aggregates in the dorsal striatum, cerebral cortex, and SNpc, accompanied by an increase in the number of dead DA-ergic neurons and worsening motor disorders [35]. It should be noted that after six months in the SNpc, in contrast to other studied structures, a decrease in the number of α-synuclein aggregates was noted, but their content in Aqp4 knockout mice was still higher than that in wild-type (WT) mice. The authors concluded that decreased AQP4 expression may exacerbate PD-like pathology, and a possible cause is the disruption of the glymphatic pathway. Some studies have also demonstrated that Aqp4 knockout mice in the PD model were characterized by an aggravated loss of tyrosine hydroxylase (TH)-positive neurons in comparison with WT mice [125,126,127]. The application of the AQP4 inhibitor TGN-020 in a PD model based on proteasome dysfunction led to a similar effect, inducing the aggravation of nigral DA-ergic neuron loss and the development of motor dysfunction [128,129].

Recent studies exploring the role of matrix metalloproteinase-9 (MMP-9)-mediated β-dystroglycan (β-DG) cleavage in the regulation of AQP4 polarity-mediated glymphatic system in PD have revealed reduced perivascular influx and efflux of CSF tracers in an MPTP-induced PD model in mice associated with impaired AQP4 polarization [130]. The application of TGN-020, an AQP4 inhibitor, evoked the aggravation of reactive astrogliosis, glymphatic drainage restriction, and the loss of nigral DA-ergic neurons in an MPTP-induced PD model. The authors demonstrated that MMP-9 and cleaved β-DG were upregulated in both MPTP-induced PD and A53T mice, while the polarized localization of β-DG and AQP4 to astrocyte endfeet was reduced. MMP-9 inhibition restored basement membrane–astrocyte endfeet–AQP4 integrity and attenuated the loss of DA-ergic neurons [130]. The results of this study demonstrated the impact of AQP4 depolarization in glymphatic dysfunction and the aggravation of PD-like pathologies and revealed the important role of MMP-9-mediated β-DG cleavage in regulating AQP4 polarization and maintaining glymphatic function. This study was not aimed at an assessment of α-synuclein accumulation; however, it can be supposed to be one of the possible mechanisms involved in the aggravation of PD-like pathologies.

Thus, these data confirm a close interaction between AQP4 dysfunction and the development of PD-like pathologies. AQP4 dysfunction can be associated with a lack of its expression or polarization and may lead to impaired glymphatic clearance of α-synuclein from the intercellular space, contributing to the development or progression of neurodegenerative processes. However, the exact mechanisms of the excretion of α-synuclein and other amyloidogenic proteins from brain tissue into the CSF remain unknown, and further research in this area is required to elucidate these mechanisms.

It is worth mentioning that not only α-synuclein pathology may be present in the brain of PD patients, amyloid-β and tau can also be observed [131]. LBs have also been found in patients with AD [132]. In a human study, certain Aqp4 SNPs were found to be associated with increased amyloid-β content in the brain [31,32,120]. It has been suggested that other amyloidogenic proteins that are less characteristic of the disease may be involved in the development of certain symptoms. For example, α-synuclein may participate in the mechanisms of neurodegeneration in the hippocampus in AD and amyloid-β accumulation in PD may contribute to the development of cognitive impairment [120,132]. Previously, there was in vitro evidence that amyloid-β can promote the fibrillization of α-synuclein. α-Synuclein, which, in turn, can lead to amyloid-β aggregation [133,134]; however, in vivo experiments failed to support the evidence of mutually aggravating the interplay between amyloid-β and α-synuclein [135]. Furthermore, tau protein is a predisposing factor for PD development. α-Synuclein and tau can assist each other in spreading and fibrilization [136]. Several studies have demonstrated that AQP4 dysfunction leads to amyloid-β and tau accumulation in animal models [38,137]. Blocking lymphatic clearance via deep cervical node ligation evoked the impairment of fluorescent tracer drainage, disturbance in the polarity of AQP4, and the accumulation of pathological forms of tau and α-synuclein in the mouse midbrain [138].

These data suggest that there is a strong need to further investigate the interplay between α-synuclein, amyloid-β, and tau [31,32,120,131,132,133,134,135,136,137,138]. AQP4 is one of the common mechanisms that impact the accumulation of all these amyloidogenic proteins [24,25,27,124]. For the development of therapeutic approaches, it is important to study the relationship of AQP4 not only with each of these proteins separately but also to evaluate them in combination.

### 3.3. Potential Mechanisms Associated with AQP4 That Contribute to Alpha-Synuclein Pathology

**Figure 1:**
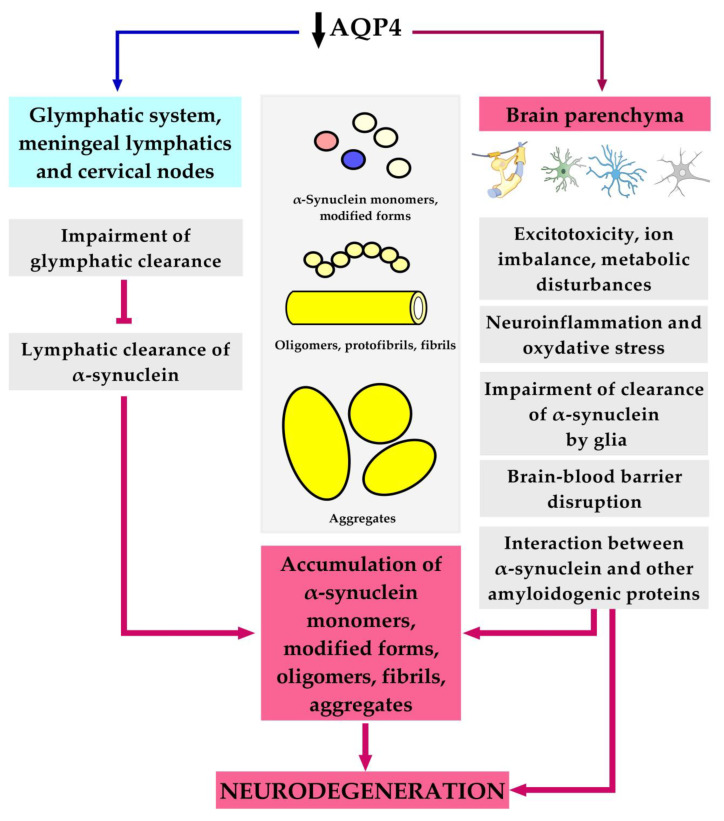
Possible AQP4-related mechanisms contributing to α-synuclein pathology.

Regarding the impact of AQP4 on brain waste clearance, the main focus is its role in the removal of metabolites (i.e., amyloidogenic proteins) from the brain parenchyma to the CSF. Since AQP4 is involved in the regulation of water homeostasis in the brain, it can be supposed that its dysfunction can lead to different consequences, such as disturbed hydrostatic pressure, water and ion balance, changes in fluid microcirculation between the paravascular, intercellular, and intracellular spaces, neurotransmitter disturbances, oxidative stress, interplay of α-synuclein and other amyloidogenic proteins, changes in astrocyte and microglial function, and BBB disruption (Figure 1). Most likely, this list is incomplete.

#### 3.3.1. AQP4 and CNS Homeostasis

The polarized expression of AQP4 plays an essential role in the mechanisms that provide stability to the water and ion environment in the CNS. It was also revealed that AQP4 dysfunction is involved in the pathogenesis of edema, tumors, and hydrocephalus [139,140,141]. A study on Aqp4 knockout mice showed that these animals were characterized by higher basal water content than WT mice [142]. It is thought that it could provoke elevated intracranial pressure, which leads to aggravated hippocampal degeneration, enlarged infarct size, hypertrophy of astrocytes, and increased mortality after ischemia–reperfusion. A recent study using diffusion MRI demonstrated that Aqp4 knockout mice are characterized by enlarged interstitial spaces and total brain volumes resulting from reduced glymphatic influx and fluid stagnation in the interstitial space [143].

AQP4 is known to be functionally and structurally associated with the volume- and thermosensitive calcium channel transient receptor potential vanilloid member-4 (TRPV4), which is also implicated in mechanisms regulating water transport and calcium homeostasis, the detection of changes in cell volume, and possibly, its regulation [144,145,146,147]. In vitro studies relating to Aqp4^-/-^ astrocytes revealed that the possible role of TRPV4 is to tune plasma membrane water permeability and protect against severe osmotic challenges due to fast AQP4-dependent water transport [146]. However, the role of TRPV4 and calcium influx from extracellular space in the regulation of cell volume is controversial, and there is evidence that TRPV4 is not essential for regulatory volume decreases after AQP4-mediated cell swelling [145,147]. Another recent study showed that mutant DA-ergic neurons raised from mutant stem cells demonstrated constitutively elevated cytosolic calcium-augmented AKT-mediated α-synuclein induction, which led to mitochondrial Ca^2+^ accumulation and dysfunction. The use of TRPV4 antagonist, AKT inhibitor, or α-synuclein knockdown induced the normalization of mitochondrial calcium levels in mutant neurons. These data suggest the importance of further investigation of such mechanisms due to their probable modulation by AQP4 and their impact on the pathogenesis of PD and other neurological disorders [148]. However, an in vivo study in a mouse MPTP model of PD revealed that adeno-associated virus (AAV)-induced TRPV4 knockdown alleviated movement deficits and nigral neurodegeneration in PD mice, whereas the upregulation of TRPV4 via the injection of a constructed AAV-TRPV4, aggravated it [149]. Moreover, this study demonstrated the involvement of TRPV4 in ER stress and inflammation, which contribute to the pathogenesis of PD [149]. This direction should be developed to determine the exact role of TRPV4 and its underlying neurodegeneration.

Moreover, a lack of AQP4 evokes the attenuation of K^+^ buffering via the Kir 4.1 channel, sex- and region-specific alterations of glutamate, serotonin, noradrenaline, DA, and some amino acids, and a decrease in glutamate uptake and glutamate transporter-1 expression in astrocytes [150,151]. The observed decrease in extracellular K^+^ content and the accumulation of extracellular glutamate leads to excitotoxicity, which, in turn, may contribute to the death of DA-ergic neurons in PD [152,153]. The selective modulation of glutamatergic neurotransmission has been shown to affect extracellular α-synuclein levels [154]; however, this effect was not due to excitotoxicity. The authors hypothesized that physiologically released α-synuclein may spread and form oligomers in the extracellular space, which contributes to the development of α-synuclein pathology; however, this hypothesis needs further investigation. Simultaneously, it was shown that α-synuclein-activated microglia produce extracellular glutamate, aggravating excitotoxicity in PD [155]. It was shown that the MPTP-induced PD model in mice was characterized by disrupted AQP4 polarization and marked changes in metabolic profile mainly composed of lipids and lipid-like molecules as well as organic acids and derivatives [130]. The restoration of AQP4 polarization led to the downregulation of neurodegeneration-associated metabolites, uremic toxins as well as secondary metabolites. At the same time, the authors revealed the upregulation of polyunsaturated fatty acids, nucleotides, phosphoglycerides, and other biosynthetic intermediates [130]. The restoration of AQP4 polarization led to the recovery of markers of anti-oxidative stress, mitophagy, and apoptosis, as well as modulators of neurotransmission and extracellular signaling. Moreover, the excitotoxic (D-aspartic acid) and neurotoxic (uremictoxins) substances were reduced [130]. Thus, this study provided a valuable impact on the knowledge of the functions of AQP4-mediated glymphatic clearance and highlighted its impact in metabolic homeostasis [130]. The aforementioned data suggest that impaired AQP4 function can evoke ionic, neurotransmitter, and metabolic disturbances, which may contribute to neurodegeneration and α-synuclein accumulation in PD.

#### 3.3.2. AQP4 and CNS Inflammation

According to much evidence, neuroinflammation is an important pathogenetic characteristic of PD. It is characterized by numerous events that impact the aggravation of DA-ergic neuron degeneration, such as microglial activation, elevated levels of proinflammatory cytokines and reactive oxygen species, and mitochondrial dysfunction [18]. Neuroinflammation is accompanied by oxidative stress, which is one of the factors inducing the formation of pathological α-synuclein and aggregation [18]. Using knockout mice, it was revealed that the lack of AQP4 is characterized by higher levels of oxidative stress in the hippocampus of ovariectomized mice [156]. Thus, deterioration of AQP4 accompanied by co-pathology (coupled with sex hormone dysfunction) may affect α-synuclein pathology development [18,157]. However, this line of research has been poorly understood and requires further investigation.

AQP4 is also thought to be implicated in CNS inflammation as well as in the regulation of immune cells in the brain; however, the data obtained are contradictory. Some studies have shown that Aqp4^−/−^ knockout mice are characterized by a reduced inflammatory cytokine response to lipopolysaccharide injection in vivo and in vitro [158,159]. In the MPP+ PD model, Aqp4 knockout mice demonstrated less prominent microglial activation than WT mice [160]. According to the authors’ opinion, these results may indicate that AQP4 plays a proinflammatory role in Parkinson’s disease, secondary to the dysregulation of astrocytic volume homeostasis. However, other studies have demonstrated the opposite effects of AQP4 deficiency. Aqp4 gene deletion in the MPTP model of PD induced a pronounced increase in proinflammatory cytokine levels, microglial inflammatory responses and aggravated loss of TH-positive neurons in comparison with WT mice [125,126,127,161]. Moreover, it has been reported that the amount of CD4(+) CD25(+) regulatory T cells was decreased in the spleen of AQP4-deficient mice, which may be, at least in part, responsible for the lack of suppression of hyperactive uncontrolled microglial and immune responses that exacerbate neurodegeneration in an MPTP-induced PD model in mice [161]. The reduction in this key population of regulatory cells could be the result of impaired generation in the thymus in the absence of AQP4 [161]. Another reason for augmented neuroinflammation is the absence of upregulation of transforming growth factor-β1 (TGF-β1), a key suppressive cytokine in the midbrain and peripheral blood of Aqp4^-/-^ mice after MPTP treatment [127]. Moreover, AQP4 deficiency has been shown not only to exacerbate neurodegeneration of DA-ergic neurons and neuroinflammation but also to reduce the difference in MPTP sensitivity between SN and VTA [126]. This, according to the authors, may be due not only to intrinsic neuronal factors but also to differences in glia in these brain regions [126]. Recently, it has been demonstrated that the glymphatic system is damaged by astrogliosis in the development of PD-like pathology [130]. Si et al. showed that AQP4 inhibition exacerbated reactive astrogliosis, restricted glymphatic drainage, and loss of dopaminergic neurons in mice with MPTP-induced Parkinson’s disease [130]. Another study showed that the development of the MPTP model of PD in Aqp4 knockout mice was characterized by astrogliosis, the inhibition of astroglial proliferation, and glial cell-derived neurotrophic factor (GDNF) synthesis was inhibited by AQP4 deficiency [162].

Thus, dysfunction of AQP4 may contribute to the pathogenesis of PD via an inflammatory response, and astrogliosis and may impede the functioning of neuroprotective mechanisms. However, these data are contradictory, and the exact role of AQP4 may depend on the specificity of the pathology in this process and remains to be defined.

Astrocytes play an important role in clearing the intercellular spaces of neurotoxic forms of α-synuclein and other aberrant proteins. Recent data have shown that astrocytes may affect not only extracellular α-synuclein but also intracellular α-synuclein [163]. Both monomeric and aggregated forms of α-synuclein can be removed via the astrocytic phagocytosis of extracellular α-synuclein and paracrine effects on the autophagic clearance of intracellular α-synuclein. The dysfunction of AQP4 may probably impair the ability of these cells to migrate, capture and degrade α-synuclein [164]. Moreover, astrocytes can play a crucial role in mediating the toxicity of α-synuclein by internalizing it upon release, which subsequently triggers an inflammatory response and other deleterious reactions [165]. Thus, it is supposed that the proper functioning of AQP4 is necessary for the modulation of inflammatory responses, astrocytic phagocytosis, and probably, for a paracrine factor-mediated mechanism of clearance.

Microglia is an essential participant in mechanisms involved in the clearance of extracellular space. Microglia can take up monomeric α-synuclein via monosialoganglioside (GM1)-dependent lipid rafts [166]. Aggregated α-synuclein can be internalized by receptor-mediated (clathrin- and calnexin-dependent) phagocytosis. Some receptors, such as Toll-like receptors TLR2, TLR4, and some others, can provide dual action via both phagocytic activity and production of proinflammatory cytokines [167]. As astrocytes can cooperate with microglia, AQP4 dysregulation may affect cytokine-mediated crosstalk between astroglia and microglia and the ability of microglia to degrade amyloidogenic proteins, particularly α-synuclein. Microgliocytes are also capable of producing exosomes containing this protein, which contribute to the further spread of α-synuclein pathology and the development of neuroinflammation [167,168]. Research devoted to investigating the interplay between AQP4 and glia is crucial for the further development of α-synuclein-targeted approaches.

#### 3.3.3. AQP4 and BBB Integrity

The BBB is an essential brain multicellular system that provides and regulates the exchange of different molecules between the brain and blood and plays a crucial role in maintaining CNS homeostasis. BBB is composed of astrocyte endfeet (enriched with AQP4) that cover blood vessels, endothelial cells and pericytes. Endothelial cells are connected by specialized tight junctions that selectively prevent the paracellular entrance of different substances from the blood to the brain [169,170]. The permeability of the BBB is not a constant characteristic; it can be dependent on the vigilance state (sleep or wakefulness) or changes related to the pathological state. Currently, many neurological disorders, including neurodegenerative and, in particular, PD, are characterized by BBB disruption [170,171]. BBB impairment can result in the entrance of blood-borne substances to the brain, edema, and the entrance of leukocytes. α-Synuclein can bidirectionally cross the BBB via endocytosis, carrier-mediated transports and nanotubes [171,172]. It is assumed that α-synuclein can impair the BBB due to its ability to affect endothelial cells and tight junctions, as well as induce gliosis and inflammation, which also can affect BBB permeability [171,173]. There is some evidence that AQP4 dysfunction can impair the BBB via detachment of astrocytic endfeet, increased fibrinogen extravasation, and altered fluid exchange [174]. Thus, both α-synuclein and AQP4 can affect BBB integrity, and there is a possibility of their synergistic effect; however, it remains unclear which is the primary action.

AQP4 is located at the interface between the brain tissue and CSF. Numerous studies have shown that the disruption of AQP4 function due to decreased expression, inhibition or mislocalization can lead to the impaired glymphatic excretion of α-synuclein from the brain parenchyma into the CSF and subsequently to the lymphatic system. On the other hand, AQP4 is known as a regulator of water homeostasis in the CNS, and we may suppose that impairment of its function could have a multidirectional damaging effect.

The mechanisms of most of these pathways are not yet fully understood.

## 4. Future Directions

Current data show that PD is a multifactorial, multisystem and variable disturbance that can have personal peculiarities in every case. This disease is a perfect candidate for the application of personalized medicine. One of the biggest challenges in its treatment is the variety of α-synuclein forms that provide multiple deleterious actions. As AQP4 has a whole-brain influence and affects many mechanisms that can be involved in neuroprotection, its modulation is suggested to be a protective approach.

Sufficient night sleep and physical activity are supposed to be the first and most obvious ways to naturally affect the brain waste clearance system. It has been postulated that the glymphatic system works most effectively during deep sleep. However, these findings were originally obtained in narcotized animals, which is a reason for criticism [175]. Nevertheless, numerous investigations have indicated an interconnection between sleep, AQP4 and glymphatic/lymphatic functions [176,177,178]. In mice, glymphatic clearance possesses endogenous circadian rhythms, with a peak during the mid-rest phase [177]. Moreover, CSF drainage from the cisterna magna to the lymph nodes demonstrated daily variation opposite to glymphatic influx. In addition, perivascular polarization of AQP4 was the highest during the rest phase. The loss of AQP4 leads to the absence of diurnal differences in glymphatic influx and drainage to the lymph nodes [177]. Sleep deprivation causes a reduction in influx efficiency along the perivascular space, disturbs AQP4 polarization and induces anxiety-like behaviors [179]. Sleep deprivation in Aqp4 knockout animals induces the accumulation of amyloid-βand tau proteins, the activation of microglia, the aggravation of neuroinflammation and synaptic protein loss in the hippocampus. These alterations were accompanied by a worsening of working memory compared to that in WT sleep-deprived mice [180]. Some genetic variants of AQP4 are associated with poor sleep quality and amyloid-β burden in humans [31]. Another study has shown that patients with chronic insomnia have decreased serum AQP4, connexin-30 (CX30) and connexin-43 (CX43). The authors suggested that it can indicate astrocyte dysfunction, which could be related to poor objective sleep quality and/or cognitive dysfunction [181]. These data indicated a close interconnection between sleep and AQP4-dependent mechanisms. Knowledge of how these physiological rhythms may impact glymphatic function and brain microenvironment is important for the development of strategies aimed at preventing adverse health outcomes, particularly neurodegeneration. One potent candidate drug that can modulate AQP4 and possesses multifaceted actions is melatonin. It was shown to relieve depressive symptoms and sleep disturbances in mice subjected to chronic, unpredictable mild stress [182]. Interestingly, melatonin treatment restored glymphatic system function and the polarization of AQP4, which were impaired in this model. It also rectified the abnormal expression of different circadian clock proteins [182]. Other links between the mechanisms of sleep regulation and AQP4 regulation were demonstrated in that gamma-aminobutiric acid (GABA), known as a “sleep mediator”, promotes glymphatic clearance in an AQP4-dependent manner via GABA_A_ receptors. Continuous theta burst stimulation can affect glymphatic clearance via GABA activation. This approach is important for the development of amyloidogenic protein-targeted treatments [183]. Thus, the studies mentioned above show the close interplay between sleep and AQP4 function and demonstrate the importance of this scientific direction. AQP4 may be a common pathogenic mechanism in neurodegenerative diseases and sleep disorders.

Another modern approach to brain waste clearance is photobiostimulation therapy (PS). This method is based on the use of nonthermal light to enhance tissue repair and waste clearance [184]. This approach has been shown to improve cognitive functions and motor behavior, decrease amyloid-β levels in the brain, and increase amyloid-β levels in the cervical lymph nodes of mice with a model of AD. The in vitro experiment showed that PS evoked an increase in BBB permeability to amyloid-β, associated with a decrease in trans-endothelial resistance and in the expression of tight junction proteins. This research suggests that PS provides BBB opening and activation of lymphatic clearance, which improves the elimination of amyloid-β from the AD mouse brain [184]. This method is a promising therapeutic approach, which explains a possible way to eliminate protein waste from the brain parenchyma. However, it remains unclear whether BBB opening can induce side effects. It can be hypothesized that PS can affect AQP4 function, as AQP4 is an important component of the BBB.

One of the first proposed ways to modulate AQP4 is through the use of pharmacological modulators. There are a number of AQP4 inhibitors, such as arylsulfonamide, acetazolamide, TGN-020, and AER-270, and one reported pharmacological facilitator, TGN-073 [185,186,187,188]. The development of these substances appears promising for the treatment of edema, fluid volume disorders and life-threatening emergency conditions. However, the effects of AQP4 inhibitors are not always reproducible [185]. In an MRI study, the AQP4 facilitator TGN-073 evoked increased brain water diffusivity, more extensive distribution, and parenchymal uptake of the Gd-DTPA tracer in TGN-073-treated rats [187]. The authors assumed that TGN-073 can improve glymphatic function in the brain and can be one more promising candidate compound that can help eliminate amyloidogenic proteins. However, the application of pharmacological AQP4 modulators may not be beneficial in the case of PD due to insufficient druggability, probable side effects owing to the region-specific distribution of AQP4, its multifactorial implication in different pathways, and the impact of co-pathologies [107,108,109,185,189]. Aging is thought to disrupt the localization of AQP4, which relocates from astrocyte endfeet [30,111,112,113]. The development of a treatment strategy aimed at correcting AQP4 localization appears to be more suitable [185,189].

Thus, we can conclude that the role of AQP4 in the development of PD and other neurodegenerative diseases is a challenging line of research, and many gaps in our knowledge concerning the mechanisms of its involvement remain unfilled and require further human and animal studies. However, as we noticed, studies devoted to the role of AQP4 in PD pathogenesis were performed mostly on Aqp4 knockout mice in MPTP- or α-synuclein-induced models of PD. In our opinion, to elucidate the role of AQP4 in PD, particularly in the mechanisms of α-synuclein accumulation, it is necessary to apply different approaches to affect AQP4 function and various animal models of PD. This is critically important for the further development of therapeutic approaches aimed at the function of AQP4 itself and/or other aforementioned factors, which can be affected by AQP4 dysfunction. Despite the evidence that all previously described AQP4-related mechanisms contributing to a-synuclein pathology can be useful as probable targets, the mechanisms of this implication are still poorly understood and even contradictory. Another unsolved question concerns the possible sex-related peculiarities of AQP4 functioning.

Due to the importance of the design of translational investigations, studying the influence of aging and other pathologies can make sense. Both AQP4 dysfunction and α-synuclein elevation may contribute to the pathogenesis of other common disturbances, such as epilepsy [190,191,192] and diabetes mellitus [193,194]. The investigation of the interconnection between these disorders and the development of PD (as well as other neurodegenerative diseases) seems to be very important for personalized medicine and treatment strategies. As AQP4 is involved in many brain functions, its modulation may act as a double-edged sword, which helps correct one disturbance and aggravates another.

## 5. Conclusions

Given the essential role of α-synuclein in the development of PD, this protein may be a target for therapeutic approaches aimed at treating PD. However, despite active efforts to develop tests for the early diagnosis of PD and different strategies to counteract α-synuclein production and propagation, PD remains incurable. The diversity of α-synuclein forms and species, as well as its intracellular and extracellular localization, make the search for and design of means to affect α-synuclein very difficult. Accumulating data have shown that disturbances in AQP4-dependent glymphatic clearance in the brain may play a crucial role in the development of PD and other neurodegenerative diseases. This scientific direction has attracted much attention; however, significant gaps in our knowledge regarding the exact role of AQP4 in this process exist. The most evident data were obtained; however, multiple mechanisms of AQP4 involvement in brain homeostasis remain in the shade. This is a challenge owing to the peculiarities of human studies, the availability of post-mortem material, and methodological issues. Another challenge is that AQP4 is multifaceted, and its state can play both protective and detrimental roles depending on conditions/co-pathologies. Nevertheless, current data on the role of AQP4 dysfunction in the accumulation of less explored α-synuclein and more explored amyloid-β and tau provide evidence that the proper functioning of AQP4 is necessary for multiple tissue and cellular mechanisms that affect brain homeostasis. Thus, further studies on the role of AQP4 in the mechanisms of neuroprotection and brain waste clearance look challenging but promising and essential for the search for AQP4-based therapeutics aimed at the treatment of PD and other neurodegenerative disorders.

## Abbreviations

AD	Alzheimer’s disease
AQP4	aquaporin-4
BBB	blood–brain barrier
CNS	central nervous system
CSF	cerebrospinal fluid
DA	dopamine
ER stress	endoplasmic reticulum stress
LBs	Lewy bodies
MPTP	1-methyl-4-phenyl-1,2,3,6-tetrahydropyridine
MRI	magnetic resonance imaging
PD	Parkinson’s disease
SN	substantia nigra
SNP	single-nucleotide polymorphism
SNpc	substantia nigra pars compacta
TH	tyrosine hydroxylase
WT	wild type

## References

[1] Ou Z., Pan J., Tang S., Duan D., Yu D., Nong H., Wang Z. (2021). Global Trends in the Incidence, Prevalence, and Years Lived with Disability of Parkinson’s Disease in 204 Countries/Territories from 1990 to 2019. Front. Public Health.

[2] Montemurro N., Aliaga N., Graff P., Escribano A., Lizana J. (2022). New Targets and New Technologies in the Treatment of Parkinson’s Disease: A Narrative Review. Int. J. Environ. Res. Public Health.

[3] Bernheimer H., Birkmayer W., Hornykiewicz O., Jellinger K., Seitelberger F. (1973). Brain Dopamine and the Syndromes of Parkinson and Huntington Clinical, Morphological and Neurochemical Correlations. J. Neurol. Sci..

[4] Postuma R.B., Berg D., Stern M., Poewe W., Olanow C.W., Oertel W., Obeso J., Marek K., Litvan I., Lang A.E. (2015). MDS Clinical Diagnostic Criteria for Parkinson’s Disease. Mov. Disord..

[5] Furukawa K., Shima A., Kihara D., Nishida A., Wada I., Sakamaki H., Yoshimura K., Terada Y., Sakato Y., Mitsuhashi M. (2022). Motor Progression and Nigrostriatal Neurodegeneration in Parkinson Disease. Ann. Neurol..

[6] Braak H., Del Tredici K. (2009). Neuroanatomy and Pathology of Sporadic Parkinson’s Disease. Adv. Anat. Embryol. Cell Biol..

[7] Del Tredici K., Braak H. (2020). To Stage, or Not to Stage. Curr. Opin. Neurobiol..

[8] Del Tredici K., Hawkes C.H., Ghebremedhin E., Braak H. (2010). Lewy Pathology in the Submandibular Gland of Individuals with Incidental Lewy Body Disease and Sporadic Parkinson’s Disease. Acta Neuropathol..

[9] Wolters E.C. (2009). Non-Motor Extranigral Signs and Symptoms in Parkinson’s Disease. Park. Relat. Disord..

[10] Kupsky W.J., Grimes M.M., Sweeting J., Bertsch R., Cote L.J. (1987). Parkinson’s Disease and Megacolon: Concentric Hyaline Inclusions (Lewy Bodies) in Enteric Ganglion Cells. Neurology.

[11] Hawkes C.H., Del Tredici K., Braak H. (2007). Parkinson’s Disease: A Dual-Hit Hypothesis. Neuropathol. Appl. Neurobiol..

[12] Jain S. (2011). Multi-Organ Autonomic Dysfunction in Parkinson Disease. Park. Relat. Disord..

[13] Hustad E., Aasly J.O. (2020). Clinical and Imaging Markers of Prodromal Parkinson’s Disease. Front. Neurol..

[14] Masuda-Suzukake M., Nonaka T., Hosokawa M., Oikawa T., Arai T., Akiyama H., Mann D.M.A., Hasegawa M. (2013). Prion-like Spreading of Pathological α-Synuclein in Brain. Brain.

[15] Chartier-Harlin M.-C., Kachergus J., Roumier C., Mouroux V., Douay X., Lincoln S., Levecque C., Larvor L., Andrieux J., Hulihan M. (2004). α-Synuclein Locus Duplication as a Cause of Familial Parkinson’s Disease. Lancet.

[16] Chen M., Mor D.E. (2023). Gut-To-Brain α-Synuclein Transmission in Parkinson’s Disease: Evidence for Prion-like Mechanisms. Int. J. Mol. Sci..

[17] Spillantini M.G., Crowther R.A., Jakes R., Hasegawa M., Goedert M. (1998). α-Synuclein in Filamentous Inclusions of Lewy Bodies from Parkinson’s Disease and Dementia with Lewy Bodies. Proc. Natl. Acad. Sci. USA.

[18] Calabresi P., Mechelli A., Natale G., Volpicelli-Daley L., Di Lazzaro G., Ghiglieri V. (2023). Alpha-Synuclein in Parkinson’s Disease and Other Synucleinopathies: From Overt Neurodegeneration back to Early Synaptic Dysfunction. Cell Death Dis..

[19] Hodara R., Norris E.H., Giasson B.I., Mishizen-Eberz A.J., Lynch D.R., Lee V.M.-Y., Ischiropoulos H. (2004). Functional Consequences of α-Synuclein Tyrosine Nitration. J. Biol. Chem..

[20] Paxinou E., Chen Q., Weisse M., Giasson B.I., Norris E.H., Rueter S.M., Trojanowski J.Q., Lee V.M.-Y., Ischiropoulos H. (2001). Induction of α-Synuclein Aggregation by Intracellular Nitrative Insult. J. Neurosci..

[21] Danzer K.M., Ruf W.P., Putcha P., Joyner D., Hashimoto T., Glabe C., Hyman B.T., McLean P.J. (2010). Heat-Shock Protein 70 Modulates Toxic Extracellular α-Synuclein Oligomers and Rescues Trans-Synaptic Toxicity. FASEB J..

[22] Ebrahimi-Fakhari D., Cantuti-Castelvetri I., Fan Z., Rockenstein E., Masliah E., Hyman B.T., McLean P.J., Unni V.K. (2011). Distinct Roles in Vivo for the Ubiquitin-Proteasome System and the Autophagy-Lysosomal Pathway in the Degradation of α-Synuclein. J. Neurosci..

[23] Riederer P., Berg D., Casadei N., Cheng F., Classen J., Dresel C., Jost W., Krüger R., Müller T., Reichmann H. (2019). α-Synuclein in Parkinson’s Disease: Causal or Bystander?. J. Neural Transm..

[24] Iliff J.J., Wang M., Liao Y., Plogg B.A., Peng W., Gundersen G.A., Benveniste H., Vates G.E., Deane R., Goldman S.A. (2012). A Paravascular Pathway Facilitates CSF Flow through the Brain Parenchyma and the Clearance of Interstitial Solutes, Including Amyloid-β. Sci. Transl. Med..

[25] Nedergaard M. (2013). Garbage Truck of the Brain. Science.

[26] Louveau A., Smirnov I., Keyes T.J., Eccles J.D., Rouhani S.J., Peske J.D., Derecki N.C., Castle D., Mandell J.W., Lee K.S. (2015). Structural and Functional Features of Central Nervous System Lymphatic Vessels. Nature.

[27] Semyachkina-Glushkovskaya O.V., Postnov D.E., Khorovodov A., Navolokin N.A., Kurthz J.H.G. (2023). Lymphatic Drainage System of the Brain: A New Player in Neuroscience. J. Evol. Biochem. Physiol..

[28] Hoshi A., Yamamoto T., Shimizu K., Ugawa Y., Nishizawa M., Takahashi H., Kakita A. (2012). Characteristics of Aquaporin Expression Surrounding Senile Plaques and Cerebral Amyloid Angiopathy in Alzheimer Disease. J. Neuropathol. Exp. Neurol..

[29] Suzuki Y., Nakamura Y., Yamada K., Igarashi H., Kasuga K., Yokoyama Y., Ikeuchi T., Nishizawa M., Kwee I.L., Nakada T. (2015). Reduced CSF Water Influx in Alzheimer’s Disease Supporting the β-Amyloid Clearance Hypothesis. PLoS ONE.

[30] Zeppenfeld D.M., Simon M., Haswell J.D., D’Abreo D., Murchison C., Quinn J.F., Grafe M.R., Woltjer R.L., Kaye J., Iliff J.J. (2017). Association of Perivascular Localization of Aquaporin-4 with Cognition and Alzheimer Disease in Aging Brains. JAMA Neurol..

[31] Rainey-Smith S.R., Mazzucchelli G.N., Villemagne V.L., Brown B.M., Porter T., Weinborn M., Bucks R.S., Milicic L., Sohrabi H.R., Taddei K. (2018). Genetic Variation in Aquaporin-4 Moderates the Relationship between Sleep and Brain Aβ-Amyloid Burden. Transl. Psychiatry.

[32] Chandra A., Farrell C., Wilson H., Dervenoulas G., De Natale E.R., Politis M. (2021). Aquaporin-4 Polymorphisms Predict Amyloid Burden and Clinical Outcome in the Alzheimer’s Disease Spectrum. Neurobiol. Aging.

[33] Hoshi A., Tsunoda A., Tada M., Nishizawa M., Ugawa Y., Kakita A. (2016). Expression of Aquaporin 1 and Aquaporin 4 in the Temporal Neocortex of Patients with Parkinson’s Disease. Brain Pathol..

[34] Ofori E., Pasternak O., Planetta P.J., Burciu R., Snyder A., Febo M., Golde T.E., Okun M.S., Vaillancourt D.E. (2015). Increased Free-Water in the Substantia Nigra of Parkinson’s Disease: A Single-Site and Multi-Site Study. Neurobiol. Aging.

[35] Cui H., Wang W., Zheng X., Xia D., Liu H., Qin C., Tian H., Teng J. (2021). Decreased AQP4 Expression Aggravates ɑ-Synuclein Pathology in Parkinson’s Disease Mice, Possibly via Impaired Glymphatic Clearance. J. Mol. Neurosci..

[36] Zhang Y., Zhang C., He X.-Z., Li Z., Meng J.-C., Mao R.-T., Li X., Xue R., Gui Q., Zhang G. (2023). Interaction between the Glymphatic System and α-Synuclein in Parkinson’s Disease. Mol. Neurobiol..

[37] Smith A.J., Duan T., Verkman A.S. (2019). Aquaporin-4 Reduces Neuropathology in a Mouse Model of Alzheimer’s Disease by Remodeling Peri-Plaque Astrocyte Structure. Acta Neuropathol. Commun..

[38] Harrison I.F., Ismail O., Machhada A., Colgan N., Ohene Y., Nahavandi P., Ahmed Z., Fisher A., Meftah S., Murray T.K. (2020). Impaired Glymphatic Function and Clearance of Tau in an Alzheimer’s Disease Model. Brain.

[39] Rosu G.-C., Catalin B., Balseanu T.A., Laurentiu M., Claudiu M., Kumar-Singh S., Daniel P. (2020). Inhibition of Aquaporin 4 Decreases Amyloid Aβ40 Drainage around Cerebral Vessels. Mol. Neurobiol..

[40] Lee Y., Choi Y., Park E.-J., Kwon S., Kim H., Lee J.Y., Lee D.S. (2020). Improvement of Glymphatic–Lymphatic Drainage of Beta-Amyloid by Focused Ultrasound in Alzheimer’s Disease Model. Sci. Rep..

[41] Nagelhus E.A., Ottersen O.P. (2013). Physiological Roles of Aquaporin-4 in Brain. Physiol. Rev..

[42] Szczygielski J., Kopańska M., Wysocka A., Oertel J. (2021). Cerebral Microcirculation, Perivascular Unit, and Glymphatic System: Role of Aquaporin-4 as the Gatekeeper for Water Homeostasis. Front. Neurol..

[43] Chen X., de Silva H.A.R., Pettenati M.J., Rao P.N., George-Hyslop P.S., Roses A.D., Xia Y., Horsburgh K., Uéda K., Saitoh T. (1995). The Human NACP/α-Synuclein Gene: Chromosome Assignment to 4q21.3–Q22 and TaqI RFLP Analysis. Genomics.

[44] Bernal-Conde L.D., Ramos-Acevedo R., Reyes-Hernández M.A., Balbuena-Olvera A.J., Morales-Moreno I.D., Argüero-Sánchez R., Schüle B., Guerra-Crespo M. (2020). Alpha-Synuclein Physiology and Pathology: A Perspective on Cellular Structures and Organelles. Front. Neurosci..

[45] Chandra S., Fornai F., Kwon H.-B., Yazdani U., Atasoy D., Liu X., Hammer R.E., Battaglia G., German D.C., Castillo P.E. (2004). Double-Knockout Mice for α- and β-Synucleins: Effect on Synaptic Functions. Proc. Natl. Acad. Sci. USA.

[46] Benskey M.J., Sellnow R.C., Sandoval I.M., Sortwell C.E., Lipton J.W., Manfredsson F.P. (2018). Silencing Alpha Synuclein in Mature Nigral Neurons Results in Rapid Neuroinflammation and Subsequent Toxicity. Front. Mol. Neurosci..

[47] .Kokhan V.S., Afanasyeva M.A., Van’kin G.I. (2012). α-Synuclein Knockout Mice Have Cognitive Impairments. Behav. Brain Res..

[48] Abeliovich A., Schmitz Y., Fariñas I., Choi-Lundberg D., Ho W.-H., Castillo P.E., Shinsky N., Verdugo J.M.G., Armanini M., Ryan A. (2000). Mice Lacking α-Synuclein Display Functional Deficits in the Nigrostriatal Dopamine System. Neuron.

[49] Tarasova T., Lytkina O.A., Goloborshcheva V.V., Skuratovskaya L.N., Antohin A.I., Ovchinnikov R.K., Kukharsky M.S. (2018). Genetic Inactivation of Alpha-Synuclein Affects Embryonic Development of Dopaminergic Neurons of the Substantia Nigra, but Not the Ventral Tegmental Area, in Mouse Brain. PeerJ.

[50] Vorobyov V., Deev A., Sukhanova I., Morozova O., Oganesyan Z., Chaprov K., Buchman V. (2022). Loss of the Synuclein Family Members Differentially Affects Baseline- and Apomorphine-Associated EEG Determinants in Single-, Double- and Triple-Knockout Mice. Biomedicines.

[51] Surguchov A., Surguchev A. (2022). Synucleins: New Data on Misfolding, Aggregation and Role in Diseases. Biomedicines.

[52] Uversky V., Eliezer D. (2009). Biophysics of Parkinsons Disease: Structure and Aggregation of α- Synuclein. Curr. Protein Pept. Sci..

[53] Singleton A.B. (2003). Alpha-Synuclein Locus Triplication Causes Parkinson’s Disease. Science.

[54] Wakabayashi K., Hayashi S., Kakita A., Yamada M., Toyoshima Y., Yoshimoto M., Takahashi H. (1998). Accumulation of α-Synuclein/NACP Is a Cytopathological Feature Common to Lewy Body Disease and Multiple System Atrophy. Acta Neuropathol..

[55] Wakabayashi K., Yoshimoto M., Tsuji S., Takahashi H. (1998). α-Synuclein Immunoreactivity in Glial Cytoplasmic Inclusions in Multiple System Atrophy. Neurosci. Lett..

[56] Alam P., Bousset L., Melki R., Otzen D.E. (2019). α-Synuclein Oligomers and Fibrils: A Spectrum of Species, a Spectrum of Toxicities. J. Neurochem..

[57] Mahul-Mellier A.-L., Burtscher J., Maharjan N., Weerens L., Croisier M., Kuttler F., Leleu M., Knott G.W., Lashuel H.A. (2020). The Process of Lewy Body Formation, rather than Simply α-Synuclein Fibrillization, Is One of the Major Drivers of Neurodegeneration. Proc. Natl. Acad. Sci. USA.

[58] Bodner R.A., Outeiro T.F., Altmann S., Maxwell M.M., Cho S.H., Hyman B.T., McLean P.J., Young A.B., Housman D.E., Kazantsev A.G. (2006). Pharmacological Promotion of Inclusion Formation: A Therapeutic Approach for Huntington’s and Parkinson’s Diseases. Proc. Natl. Acad. Sci. USA.

[59] Tanaka M., Kim Y.M., Lee G., Junn E., Iwatsubo T., Mouradian M.M. (2004). Aggresomes Formed by Alpha-Synuclein and Synphilin-1 Are Cytoprotective. J. Biol. Chem..

[60] Du X., Xie X., Liu R. (2020). The Role of α-Synuclein Oligomers in Parkinson’s Disease. Int. J. Mol. Sci..

[61] Arawaka S., Sato H., Sasaki A., Koyama S., Kato T. (2017). Mechanisms Underlying Extensive Ser129-Phosphorylation in α-Synuclein Aggregates. Acta Neuropathol. Commun..

[62] Zenko D., Marsh J., Castle A.R., Lewin R., Fischer R., Tofaris G.K. (2023). Monitoring α-Synuclein Ubiquitination Dynamics Reveals Key Endosomal Effectors Mediating Its Trafficking and Degradation. Sci. Adv..

[63] Matsui H., Ito S., Matsui H., Itô J., Gabdulkhaev R., Hirose M., Yamanaka T., Koyama A., Kato T., Tanaka M. (2023). Phosphorylation of α-Synuclein at T64 Results in Distinct Oligomers and Exerts Toxicity in Models of Parkinson’s Disease. Proc. Natl. Acad. Sci. USA.

[64] Rott R., Szargel R., Shani V., Hamza H., Savyon M., Abd Elghani F., Bandopadhyay R., Engelender S. (2017). SUMOylation and Ubiquitination Reciprocally Regulate α-Synuclein Degradation and Pathological Aggregation. Proc. Natl. Acad. Sci. USA.

[65] Chen L., Periquet M., Wang X., Negro A., McLean P.J., Hyman B.T., Feany M.B. (2009). Tyrosine and Serine Phosphorylation of α-Synuclein Have Opposing Effects on Neurotoxicity and Soluble Oligomer Formation. J. Clin. Investig..

[66] Negro A., Brunati A.M., Donella-Deana A., Massimino M.L., Pinna L.A. (2001). Multiple Phosphorylation of α-Synuclein by Protein Tyrosine Kinase Syk Prevents Eosin-Induced Aggregation. FASEB J..

[67] Machado R., Miranda H.V., Francelle L., Pinho R., Szegő É.M., Martinho R., Munari F., Lázaro D.F., Moniot S., Guerreiro P. (2017). The Mechanism of Sirtuin 2–Mediated Exacerbation of Alpha-Synuclein Toxicity in Models of Parkinson Disease. PLoS Biol..

[68] Ramalingam N., Jin S.-X., Moors T.E., Fonseca-Ornelas L., Shimanaka K., Lei S., Cam H.P., Watson A.H., Brontesi L., Ding L. (2023). Dynamic Physiological α-Synuclein S129 Phosphorylation Is Driven by Neuronal Activity. Npj Park. Dis..

[69] Isonaka R., Goldstein D.S., Zhu W., Yoon E., Ehrlich D., Schindler A.B., Kokkinis A., Sabir M.S., Scholz S.W., Bandrés-Ciga S. (2021). α-Synuclein Deposition in Sympathetic Nerve Fibers in Genetic Forms of Parkinson’s Disease. Mov. Disord..

[70] Delenclos M., Trendafilova T., Mahesh D., Baine A.M., Moussaud S., Yan I.K., Patel T., McLean P.J. (2017). Investigation of Endocytic Pathways for the Internalization of Exosome-Associated Oligomeric Alpha-Synuclein. Front. Neurosci..

[71] Rodriguez L., Marano M.M., Tandon A. (2018). Import and Export of Misfolded α-Synuclein. Front. Neurosci..

[72] Konno T., Ross O.A., Puschmann A., Dickson D.W., Wszolek Z.K. (2016). Autosomal Dominant Parkinson’s Disease Caused by SNCA Duplications. Park. Relat. Disord..

[73] Tran J., Anastacio H., Bardy C. (2020). Genetic Predispositions of Parkinson’s Disease Revealed in Patient-Derived Brain Cells. Npj Park. Dis..

[74] van Rooijen B.D., Claessens M.M.A.E., Subramaniam V. (2010). Membrane Permeabilization by Oligomeric α-Synuclein: In Search of the Mechanism. PLoS ONE.

[75] Van Den Berge N., Ferreira N., Mikkelsen T.W., Alstrup A.K.O., Tamgüney G., Karlsson P., Terkelsen A.J., Nyengaard J.R., Jensen P.H., Borghammer P. (2021). Ageing Promotes Pathological Alpha-Synuclein Propagation and Autonomic Dysfunction in Wild-Type Rats. Brain.

[76] Rodger A., ALNasser M.N., Carter W.G. (2023). Are Therapies That Target α-Synuclein Effective at Halting Parkinson’s Disease Progression? A Systematic Review. Int. J. Mol. Sci..

[77] Hmila I., Sudhakaran I.P., Ghanem S.S., Vaikath N.N., Poggiolini I., Abdesselem H., El-Agnaf O.M.A. (2022). Inhibition of α-Synuclein Seeding-Dependent Aggregation by SsDNA Aptamers Specific to C-Terminally Truncated α-Synuclein Fibrils. ACS Chem. Neurosci..

[78] Alam P., Holst M.R., Lauritsen L., Nielsen J., Simone, Jensen P.H., Brewer J.R., Otzen D.E., Nielsen M. (2022). Polarized α-Synuclein Trafficking and Transcytosis across Brain Endothelial Cells via Rab7-Decorated Carriers. Fluids Barriers CNS.

[79] Price D.L., Khan A., Angers R., Cárdenas P., Prato M.K., Bani M., Bonhaus D.W., Citron M., Biere A. (2023). In Vivo Effects of the Alpha-Synuclein Misfolding Inhibitor Minzasolmin Supports Clinical Development in Parkinson’s Disease. Npj Park. Dis..

[80] Ekimova I.V., Plaksina D.V., Pastukhov Y.F., Lapshina K.V., Lazarev V.F., Mikhaylova E.R., Polonik S.G., Pani B., Margulis B.A., Guzhova I.V. (2018). New HSF1 Inducer as a Therapeutic Agent in a Rodent Model of Parkinson’s Disease. Exp. Neurol..

[81] Daniels M.J., Nourse J.P., Kim H.-N., Sainati V., Schiavina M., Murrali M.G., Pan B., Ferrie J.J., Haney C.O., Moons R. (2019). Cyclized NDGA Modifies Dynamic α-Synuclein Monomers Preventing Aggregation and Toxicity. Sci. Rep..

[82] Xu B., Chen J., Liu Y. (2022). Curcumin Interacts with α-Synuclein Condensates to Inhibit Amyloid Aggregation under Phase Separation. ACS Omega.

[83] Lee J., Sung K.W., Bae E.J., Yoon D.W., Kim D., Lee J.S., Park D., Park D.Y., Mun S.R., Kwon S.C. (2023). Targeted Degradation of ⍺-Synuclein Aggregates in Parkinson’s Disease Using the AUTOTAC Technology. Mol. Neurodegener..

[84] Cui H., Norrbacka S., Myöhänen T.T. (2022). Prolyl Oligopeptidase Acts as a Link between Chaperone-Mediated Autophagy and Macroautophagy. Biochem. Pharmacol..

[85] Badaut J., Lasbennes F., Magistretti P.J., Regli L. (2002). Aquaporins in Brain: Distribution, Physiology, and Pathophysiology. J. Cereb. Blood Flow Metab..

[86] Preston G.M., Carroll T.P., Guggino W.B., Agre P. (1992). Appearance of Water Channels in Xenopus Oocytes Expressing Red Cell CHIP28 Protein. Science.

[87] Benga G. (2003). Birth of Water Channel Proteins—The Aquaporins. Cell Biol. Int..

[88] Hasegawa H., Ma T., Skach W., Matthay M.A., Verkman A.S. (1994). Molecular Cloning of a Mercurial-Insensitive Water Channel Expressed in Selected Water-Transporting Tissues. J. Biol. Chem..

[89] Jung J.S., Bhat R.V., Preston G.M., Guggino W.B., Baraban J.M., Agre P. (1994). Molecular Characterization of an Aquaporin CDNA from Brain: Candidate Osmoreceptor and Regulator of Water Balance. Proc. Natl. Acad. Sci. USA.

[90] Yang B., Ma T., Verkman A.S. (1995). CDNA Cloning, Gene Organization, and Chromosomal Localization of a Human Mercurial Insensitive Water Channel. J. Biol. Chem..

[91] Gleiser C., Wagner A., Fallier-Becker P., Wolburg H., Hirt B., Mack A. (2016). Aquaporin-4 in Astroglial Cells in the CNS and Supporting Cells of Sensory Organs—A Comparative Perspective. Int. J. Mol. Sci..

[92] Verkman A.S., Mitra A.K. (2000). Structure and Function of Aquaporin Water Channels. Am. J. Physiol.-Ren. Physiol..

[93] Yang B., Brown D., Verkman A.S. (1996). The Mercurial Insensitive Water Channel (AQP-4) Forms Orthogonal Arrays in Stably Transfected Chinese Hamster Ovary Cells. J. Biol. Chem..

[94] Hartwig W., Wolburg-Buchholz K., Fallier-Becker P., Noell S., Mack A.F. (2011). Structure and Functions of Aquaporin-4-Based Orthogonal Arrays of Particles. Int. Rev. Cell Mol. Biol..

[95] De Bellis M., Cibelli A., Mola M.G., Pisani F., Barile B., Mastrodonato M., Banitalebi S., Amiry-Moghaddam M., Abbrescia P., Frigeri A. (2020). Orthogonal Arrays of Particle Assembly Are Essential for Normal Aquaporin-4 Expression Level in the Brain. Glia.

[96] Crosbie H.R., Dovico S.A., Flanagan J.D., Chamberlain J.S., Ownby C.L., Campbell K.P. (2002). Characterization of Aquaporin-4 in Muscle and Muscular Dystrophy. FASEB J..

[97] Amiry-Moghaddam M., Frydenlund D.S., Ottersen O.P. (2004). Anchoring of Aquaporin-4 in Brain: Molecular Mechanisms and Implications for the Physiology and Pathophysiology of Water Transport. Neuroscience.

[98] Smith A.J., Jin B.-J., Ratelade J., Verkman A.S. (2014). Aggregation State Determines the Localization and Function of M1- and M23-Aquaporin-4 in Astrocytes. J. Cell Biol..

[99] Jin B.-J., Rossi A., Verkman A.S. (2011). Model of Aquaporin-4 Supramolecular Assembly in Orthogonal Arrays Based on Heterotetrameric Association of M1-M23 Isoforms. Biophys. J..

[100] Pisani F., Rossi A., Nicchia G.P., Svelto M., Frigeri A. (2011). Translational Regulation Mechanisms of Aquaporin-4 Supramolecular Organization in Astrocytes. Glia.

[101] De Bellis M., Pisani F., Mola M.G., Rosito S., Simone L., Buccoliero C., Trojano M., Nicchia G.P., Svelto M., Frigeri A. (2017). Translational Readthrough Generates New Astrocyte AQP4 Isoforms That Modulate Supramolecular Clustering, Glial Endfeet Localization, and Water Transport. Glia.

[102] Pati R., Palazzo C., Valente O., Abbrescia P., Messina R., Surdo N.C., Lefkimmiatis K., Signorelli F., Nicchia G.P., Frigeri A. (2022). The Readthrough Isoform AQP4ex Is Constitutively Phosphorylated in the Perivascular Astrocyte Endfeet of Human Brain. Biomolecules.

[103] Verkman A.S., Binder D.K., Bloch O., Auguste K., Papadopoulos M.C. (2006). Three Distinct Roles of Aquaporin-4 in Brain Function Revealed by Knockout Mice. Biochim. Biophys. Acta (BBA)—Biomembr..

[104] Kong H., Fan Y., Xie J., Ding J., Sha L., Shi X., Sun X., Hu G. (2008). AQP4 Knockout Impairs Proliferation, Migration and Neuronal Differentiation of Adult Neural Stem Cells. J. Cell Sci..

[105] Xie L., Kang H., Xu Q., Chen M.J., Liao Y., Thiyagarajan M., O’Donnell J., Christensen D.J., Nicholson C., Iliff J.J. (2013). Sleep Drives Metabolite Clearance from the Adult Brain. Science.

[106] Iliff J.J., Lee H., Yu M., Feng T., Logan J., Nedergaard M., Benveniste H. (2013). Brain-Wide Pathway for Waste Clearance Captured by Contrast-Enhanced MRI. J. Clin. Investig..

[107] Hoddevik E.H., Khan F.H., Rahmani S., Ottersen O.P., Boldt H.B., Amiry-Moghaddam M. (2016). Factors Determining the Density of AQP4 Water Channel Molecules at the Brain–Blood Interface. Brain Struct. Funct..

[108] Hubbard J.A., Hsu M.S., Seldin M.M., Binder D.K. (2015). Expression of the Astrocyte Water Channel Aquaporin-4 in the Mouse Brain. ASN Neuro.

[109] Prydz A., Stahl K., Puchades M., Davarpaneh N., Nadeem M., Ottersen O.P., Gundersen V., Amiry-Moghaddam M. (2017). Subcellular Expression of Aquaporin-4 in Substantia Nigra of Normal and MPTP-Treated Mice. Neuroscience.

[110] Azam S., Haque M.E., Balakrishnan R., Kim I.-S., Choi D.-K. (2021). The Ageing Brain: Molecular and Cellular Basis of Neurodegeneration. Front. Cell Dev. Biol..

[111] Simon M., Wang M.X., Ismail O., Braun M., Schindler A.G., Reemmer J., Wang Z., Haveliwala M.A., O’Boyle R.P., Han W.Y. (2022). Loss of Perivascular Aquaporin-4 Localization Impairs Glymphatic Exchange and Promotes Amyloid β Plaque Formation in Mice. Alzheimer’s Res. Ther..

[112] Kress B.T., Iliff J.J., Xia M., Wang M., Wei H.S., Zeppenfeld D., Xie L., Kang H., Xu Q., Liew J.A. (2014). Impairment of Paravascular Clearance Pathways in the Aging Brain. Ann. Neurol..

[113] Duncombe J., Lennen R.J., Jansen M.A., Marshall I., Wardlaw J.M., Horsburgh K. (2017). Ageing Causes Prominent Neurovascular Dysfunction Associated with Loss of Astrocytic Contacts and Gliosis. Neuropathol. Appl. Neurobiol..

[114] Owasil R., O’Neill R., Keable A., Nimmo J., MacGregor Sharp M., Kelly L., Saito S., Simpson J.E., Weller R.O., Smith C. (2020). The Pattern of AQP4 Expression in the Ageing Human Brain and in Cerebral Amyloid Angiopathy. Int. J. Mol. Sci..

[115] Gupta R.K., Kanungo M. (2011). Glial Molecular Alterations with Mouse Brain Development and Aging: Up-Regulation of the Kir4.1 and Aquaporin-4. AGE.

[116] Bronzuoli M.R., Facchinetti R., Valenza M., Cassano T., Steardo L., Scuderi C. (2019). Astrocyte Function Is Affected by Aging and Not Alzheimer’s Disease: A Preliminary Investigation in Hippocampi of 3xTg-AD Mice. Front. Pharmacol..

[117] Benveniste H., Liu X., Koundal S., Sanggaard S., Lee H., Wardlaw J. (2019). The Glymphatic System and Waste Clearance with Brain Aging: A Review. Gerontology.

[118] Thamizh Thenral S., Vanisree A.J. (2011). Peripheral Assessment of the Genes AQP4, PBP and TH in Patients with Parkinson’s Disease. Neurochem. Res..

[119] Ofori E., Krismer F., Burciu R.G., Pasternak O., McCracken J.L., Lewis M.M., Du G., McFarland N.R., Okun M.S., Poewe W. (2017). Free Water Improves Detection of Changes in the Substantia Nigra in Parkinsonism: A Multisite Study. Mov. Disord..

[120] Fang Y., Dai S., Jin C., Si X., Gu L., Song Z., Gao T., Chen Y., Yan Y., Yin X. (2022). Aquaporin-4 Polymorphisms Are Associated with Cognitive Performance in Parkinson’s Disease. Front. Aging Neurosci..

[121] Sun X., Tian Q., Yang Z., Li Y., Li C., Hou B., Xie A. (2023). Association of AQP4 Single Nucleotide Polymorphisms (Rs335929 and Rs2075575) with Parkinson’s Disease: A Case-Control Study. Neurosci. Lett..

[122] Jiang M., Fang Y., Dai S., Su X., Wang Z., Tang J., Gao T., Liu Y., Song Z., Pu J. (2023). The Effects of *AQP4* Rs162009 on Resting-State Brain Activity in Parkinson’s Disease. CNS Neurosci. Ther..

[123] Si X., Guo T., Wang Z., Fang Y., Gu L., Cao L., Yang W., Gao T., Song Z., Tian J. (2022). Neuroimaging Evidence of Glymphatic System Dysfunction in Possible REM Sleep Behavior Disorder and Parkinson’s Disease. Npj Park. Dis..

[124] Tamtaji O.M., Behnam M., Pourattar M.A., Jafarpour H., Asemi Z. (2019). Aquaporin 4: A Key Player in Parkinson’s Disease. J. Cell. Physiol..

[125] Sun H., Liang R., Yang B.Z., Zhou Y., Liu M., Fang F., Ding J.L., Fan Y.-Z., Hu G. (2016). Aquaporin-4 Mediates Communication between Astrocyte and Microglia: Implications of Neuroinflammation in Experimental Parkinson’s Disease. Neuroscience.

[126] Zhang J., Yang B., Sun H., Zhou Y., Liu M., Ding J., Fang F., Fan Y., Hu G. (2016). Aquaporin-4 Deficiency Diminishes the Differential Degeneration of Midbrain Dopaminergic Neurons in Experimental Parkinson’s Disease. Neurosci. Lett..

[127] Xue X., Zhang W., Zhu J., Chen X., Zhou S., Xu Z., Hu G., Su C. (2019). Aquaporin-4 Deficiency Reduces TGF-β1 in Mouse Midbrains and Exacerbates Pathology in Experimental Parkinson’s Disease. J. Cell. Mol. Med..

[128] Lapshina K.V., Abramova Y.Y., Guzeev M.A., Ekimova I.V. (2022). TGN-020, an Inhibitor of the Water Channel Aquaporin-4, Accelerates Nigrostriatal Neurodegeneration in the Rat Model of Parkinson’s Disease. J. Evol. Biochem. Physiol..

[129] Lapshina K.V., Khanina M.V., Kaismanova M.P., Ekimova I.V. (2023). Pharmacological Inhibition of AQP4 Water Channel Activity Aggravates of Alpha-Synuclein Pathology in the Substantia Nigra in a Rat Model of Parkinson’s Disease. Evol. Biochem. Physiol..

[130] Si X., Dai S., Fang Y., Tang J., Wang Z., Li Y., Song Z., Chen Y., Liu Y., Zhao G. Matrix Metalloproteinase-9 Inhibition Prevents Aquaporin-4 Depolarization-Mediated Glymphatic Dysfunction in Parkinson’s Disease. J. Adv. Res..

[131] Irwin D.J., Lee V.M.-Y., Trojanowski J.Q. (2013). Parkinson’s Disease Dementia: Convergence of α-Synuclein, Tau and Amyloid-β Pathologies. Nat. Rev. Neurosci..

[132] Chung E.J., Babulal G.M., Monsell S.E., Cairns N.J., Roe C.M., Morris J.C. (2015). Clinical Features of Alzheimer Disease with and without Lewy Bodies. JAMA Neurol..

[133] Vadukul D.M., Papp M., Thrush R.J., Wang J., Jin Y., Arosio P., Aprile F.A. (2023). α-Synuclein Aggregation Is Triggered by Oligomeric Amyloid-β 42 via Heterogeneous Primary Nucleation. J. Am. Chem. Soc..

[134] Chau E., Kim J.R. (2022). α-Synuclein-Assisted Oligomerization of β-Amyloid (1–42). Arch. Biochem. Biophys..

[135] Bachhuber T., Katzmarski N., McCarter J.F., Loreth D., Tahirovic S., Kamp F., Abou-Ajram C., Nuscher B., Serrano-Pozo A., Müller A. (2015). Inhibition of Amyloid-β Plaque Formation by α-Synuclein. Nat. Med..

[136] Pan L., Li C., Meng L., Tian Y., He M., Yuan X., Zhang G., Zhang Z., Xiong J., Chen G. (2022). Tau Accelerates α-Synuclein Aggregation and Spreading in Parkinson’s Disease. Brain J. Neurol..

[137] Iliff J.J., Chen M.J., Plog B.A., Zeppenfeld D.M., Soltero M., Yang L., Singh I., Deane R., Nedergaard M. (2014). Impairment of Glymphatic Pathway Function Promotes Tau Pathology after Traumatic Brain Injury. J. Neurosci..

[138] Zou W., Pu T., Feng W., Lu M., Zheng Y., Du R., Xiao M., Hu G. (2019). Blocking Meningeal Lymphatic Drainage Aggravates Parkinson’s Disease-like Pathology in Mice Overexpressing Mutated α-Synuclein. Transl. Neurodegener..

[139] Papadopoulos M.C., Verkman A.S. (2007). Aquaporin-4 and Brain Edema. Pediatr. Nephrol..

[140] Maugeri R., Schiera G., Di Liegro C., Fricano A., Iacopino D., Di Liegro I. (2016). Aquaporins and Brain Tumors. Int. J. Mol. Sci..

[141] Zhao Z., He J., Chen Y., Wang Y., Wang C., Tan C., Liao J., Xiao G. (2022). The Pathogenesis of Idiopathic Normal Pressure Hydrocephalus Based on the Understanding of AQP1 and AQP4. Front. Mol. Neurosci..

[142] Zeng X., Xie L., Liang R., Sun X., Fan Y., Hu G. (2012). AQP4 Knockout Aggravates Ischemia/Reperfusion Injury in Mice. CNS Neurosci. Ther..

[143] Gomolka R., Hablitz L.M., Mestre H., Giannetto M., Du T., Hauglund N.L., Xie L., Peng W., López Martínez P., Nedergaard M. (2023). Loss of Aquaporin-4 Results in Glymphatic System Dysfunction via Brain-Wide Interstitial Fluid Stagnation. eLife.

[144] Jo A.O., Ryskamp D.A., Phuong T.T.T., Verkman A.S., Yarishkin O., MacAulay N., Kriaj D. (2015). TRPV4 and AQP4 Channels Synergistically Regulate Cell Volume and Calcium Homeostasis in Retinal Muller Glia. J. Neurosci..

[145] Toft-Bertelsen T.L., Larsen B.R., MacAulay N. (2018). Sensing and Regulation of Cell Volume—We Know so Much and yet Understand so Little: TRPV4 as a Sensor of Volume Changes but Possibly without a Volume-Regulatory Role?. Channels.

[146] Barile B., Mola M.G., Formaggio F., Saracino E., Cibelli A., Gargano C.D., Mogni G., Frigeri A., Caprini M., Benfenati V. (2023). AQP4-Independent TRPV4 Modulation of Plasma Membrane Water Permeability. Front. Cell. Neurosci..

[147] Mola M.G., Sparaneo A., Gargano C.D., Spray D.C., Svelto M., Frigeri A., Scemes E., Nicchia G.P. (2015). The Speed of Swelling Kinetics Modulates Cell Volume Regulation and Calcium Signaling in Astrocytes: A Different Point of View on the Role of Aquaporins. Glia.

[148] Sun X., Kong J., Dong S., Kato H., Satō H., Hirofuji Y., Ito Y., Wang L., Kato T.A., Torio M. (2023). TRPV4-Mediated ca^2+^ Deregulation Causes Mitochondrial Dysfunction via the AKT/α-Synuclein Pathway in Dopaminergic Neurons. FASEB BioAdv..

[149] Liu N., Bai L., Lu Z., Gu R., Zhao D., Yan F., Bai J. (2022). TRPV4 Contributes to ER Stress and Inflammation: Implications for Parkinson’s Disease. J. Neuroinflamm..

[150] Fan Y., Zhang J., Sun X., Gao L., Zeng X., Ding J.-H., Cao C., Niu L., Hu G. (2005). Sex- and Region-Specific Alterations of Basal Amino Acid and Monoamine Metabolism in the Brain of Aquaporin-4 Knockout Mice. J. Neurosci. Res..

[151] Ohno Y., Kunisawa N., Shimizu S. (2021). Emerging Roles of Astrocyte Kir4.1 Channels in the Pathogenesis and Treatment of Brain Diseases. Int. J. Mol. Sci..

[152] Vandebroek A., Yasui M. (2020). Regulation of AQP4 in the Central Nervous System. Int. J. Mol. Sci..

[153] Zhang Y., Tan F., Xu P., Qu S. (2016). Recent Advance in the Relationship between Excitatory Amino Acid Transporters and Parkinson’s Disease. Neural Plast..

[154] Yamada K., Iwatsubo T. (2018). Extracellular α-Synuclein Levels Are Regulated by Neuronal Activity. Mol. Neurodegener..

[155] dos -Santos-Pereira M., Acuña L., Hamadat S., Rocca J., González-Lizárraga F., Chehín R., Sepulveda-Diaz J.E., Aparecida E., Raisman-Vozari R., Michel P. (2018). Microglial Glutamate Release Evoked by α-Synuclein Aggregates Is Prevented by Dopamine. Glia.

[156] Liu L., Lu Y., Kong H., Li L., Marshall C., Xiao M., Ding J., Gao J., Hu G. (2011). Aquaporin-4 Deficiency Exacerbates Brain Oxidative Damage and Memory Deficits Induced by Long-Term Ovarian Hormone Deprivation and D-Galactose Injection. Int. J. Neuropsychopharmacol..

[157] Won S.J., Fong R., Butler N., Sanchez J., Zhang Y., Wong C., Tambou Nzoutchoum O., Huynh A., Pan J., Swanson R.A. (2022). Neuronal Oxidative Stress Promotes α-Synuclein Aggregation in Vivo. Antioxidants.

[158] Li L., Zhang H., Varrin-Doyer M., Zamvil S.S., Verkman A.S. (2011). Proinflammatory Role of Aquaporin-4 in Autoimmune Neuroinflammation. FASEB J..

[159] Dai W., Yan J., Chen G., Hu G., Zhou X., Zeng X. (2018). AQP4-Knockout Alleviates the Lipopolysaccharide-Induced Inflammatory Response in Astrocytes via SPHK1/MAPK/AKT Signaling. Int. J. Mol. Med..

[160] Prydz A., Stahl K., Zahl S., Skauli N., Skare Ø., Ottersen O.P., Amiry-Moghaddam M. (2020). Pro-Inflammatory Role of AQP4 in Mice Subjected to Intrastriatal Injections of the Parkinsonogenic Toxin MPP+. Cells.

[161] Chi Y., Fan Y., He L., Liu W., Wen X., Zhou S., Wang X., Zhang C., Kong H., Sonoda L. (2011). Novel Role of Aquaporin-4 in CD4+ CD25+ T Regulatory Cell Development and Severity of Parkinson’s Disease. Aging Cell.

[162] Fan Y., Kong H., Shi X., Sun X., Ding J., Wu J., Hu G. (2008). Hypersensitivity of Aquaporin 4-Deficient Mice to 1-Methyl-4-Phenyl-1,2,3,6-Tetrahydropyrindine and Astrocytic Modulation. Neurobiol. Aging.

[163] Yang Y., Song J.-J., Choi Y.R., Kim S., Seok M.-J., Wulansari N., Darsono W.H.W., Kwon O.-C., Chang M.-Y., Park S.M. (2022). Therapeutic Functions of Astrocytes to Treat α-Synuclein Pathology in Parkinson’s Disease. Proc. Natl. Acad. Sci. USA.

[164] Saadoun S., Papadopoulos M.C., Watanabe H., Yan D., Manley G.T., Verkman A.S. (2005). Involvement of Aquaporin-4 in Astroglial Cell Migration and Glial Scar Formation. J. Cell Sci..

[165] Domingues R., Sant’Anna R., da Fonseca A.C.C., Robbs B.K., Foguel D., Outeiro T.F. (2022). Extracellular Alpha-Synuclein: Sensors, Receptors, and Responses. Neurobiol. Dis..

[166] Park J.Y., Kim K.S., Lee S., Ryu J., Chung K.C., Choo Y., Jou I., Kim J., Park S.M. (2009). On the Mechanism of Internalization of α-Synuclein into Microglia: Roles of Ganglioside GM1 and Lipid Raft. J. Neurochem..

[167] Yi S., Wang L., Wang H., Ho M.S., Zhang S. (2022). Pathogenesis of α-Synuclein in Parkinson’s Disease: From a Neuron-Glia Crosstalk Perspective. Int. J. Mol. Sci..

[168] Li J., Zhou Y., Wang Y., Fong H., Murray T.M., Zhang J. (2007). Identification of Proteins Involved in Microglial Endocytosis of α-Synuclein. J. Proteome Res..

[169] Nicchia G.P., Nico B., Camassa L.M.A., Mola M.G., Loh N., Dermietzel R., Spray D.C., Svelto M., Frigeri A. (2004). The Role of Aquaporin-4 in the Blood–Brain Barrier Development and Integrity: Studies in Animal and Cell Culture Models. Neuroscience.

[170] Wu D., Chen Q., Chen X., Han F., Chen Z., Wang Y. (2023). The Blood–Brain Barrier: Structure, Regulation, and Drug Delivery. Signal Transduct. Target. Ther..

[171] Bogale T.A., Faustini G., Longhena F., Mitola S., Pizzi M., Bellucci A. (2021). Alpha-Synuclein in the Regulation of Brain Endothelial and Perivascular Cells: Gaps and Future Perspectives. Front. Immunol..

[172] Duan Q., Zhang Q., Nie K., Huang R.-Z., Yang J., He P., Tie Z., Huang H., Ma G., Zhang Y. (2023). Myo1d Promotes Alpha-Synuclein Transfer from Brain Microvascular Endothelial Cells to Pericytes through Tunneling Nanotubes. iScience.

[173] Dohgu S., Takata F., Matsumoto J., Kimura I., Yamauchi A., Kataoka Y. (2019). Monomeric α-Synuclein Induces Blood–Brain Barrier Dysfunction through Activated Brain Pericytes Releasing Inflammatory Mediators in Vitro. Microvasc. Res..

[174] Zhou J., Kong H., Hua X., Xiao M., Ding J., Hu G. (2008). Altered Blood–Brain Barrier Integrity in Adult Aquaporin-4 Knockout Mice. NeuroReport.

[175] Ferris C.F. (2021). Rethinking the Conditions and Mechanism for Glymphatic Clearance. Front. Neurosci..

[176] Massey A., Boag M.K., Magnier A., Bispo D.P.C.F., Khoo T.K., Pountney D.L. (2022). Glymphatic System Dysfunction and Sleep Disturbance May Contribute to the Pathogenesis and Progression of Parkinson’s Disease. Int. J. Mol. Sci..

[177] Hablitz L.M., Plá V., Giannetto M., Vinitsky H.S., Stæger F.F., Metcalfe T., Nguyen R., Benrais A., Nedergaard M. (2020). Circadian Control of Brain Glymphatic and Lymphatic Fluid Flow. Nat. Commun..

[178] Postnov D., Semyachkina-Glushkovskaya O.V., Litvinenko E., Kurths J., Penzel T. (2023). Mechanisms of Activation of Brain’s Drainage during Sleep: The Nightlife of Astrocytes. Cells.

[179] Liu D.-X., He X., Wu D., Zhang Q., Yang C., Liang F.-Y., He X.-F., Dai G.-Y., Pei Z., Lan Y. (2017). Continuous Theta Burst Stimulation Facilitates the Clearance Efficiency of the Glymphatic Pathway in a Mouse Model of Sleep Deprivation. Neurosci. Lett..

[180] Zhang R., Liu Y., Chen Y., Li Q., Marshall C., Wu T., Hu G., Xiao M. (2019). Aquaporin 4 Deletion Exacerbates Brain Impairments in a Mouse Model of Chronic Sleep Disruption. CNS Neurosci. Ther..

[181] Yang S., Kong X.-Y., Hu T., Ge Y., Li X., Chen J., He S., Zhang P., Chen G. (2022). Aquaporin-4, Connexin-30, and Connexin-43 as Biomarkers for Decreased Objective Sleep Quality And/or Cognition Dysfunction in Patients with Chronic Insomnia Disorder. Front. Psychiatry.

[182] Yao D., Li R., Hao J., Huang H., Wang X., Ran L., Fang Y., He Y., Wang W., Liu X. (2023). Melatonin Alleviates Depression-like Behaviors and Cognitive Dysfunction in Mice by Regulating the Circadian Rhythm of AQP4 Polarization. Transl. Psychiatry.

[183] Wu C.-C., Zhang Q., Feng Y., Zhang N., Liu Q., Ou Z., Lin T., Ding Q., Li G., Pei Z. (2023). GABA Promotes Interstitial Fluid Clearance in an AQP4-Dependent Manner by Activating the GABA_A_R. J. Neurochem..

[184] Semyachkina-Glushkovskaya O.V., Abdurashitov A.S., Klimova M., Dubrovsky A., Shirokov A., Fomin A.S., Terskov A., Agranovich I., Mamedova A., Khorovodov A. (2020). Photostimulation of Cerebral and Peripheral Lymphatic Functions. Transl. Biophotonics.

[185] Salman M.M., Kitchen P., Yool A.J., Bill R.M. (2022). Recent Breakthroughs and Future Directions in Drugging Aquaporins. Trends Pharmacol. Sci..

[186] Huber V.J., Igarashi H., Ueki S., Kwee I.L., Nakada T. (2018). Aquaporin-4 Facilitator TGN-073 Promotes Interstitial Fluid Circulation within the Blood–Brain Barrier. NeuroReport.

[187] Alghanimy A., Martin C., Gallagher L., Holmes W.M. (2023). The Effect of a Novel AQP4 Facilitator, TGN-073, on Glymphatic Transport Captured by Diffusion MRI and DCE-MRI. PLoS ONE.

[188] Verghese J.P., Terry A., de Natale E.R., Politis M. (2022). Research Evidence of the Role of the Glymphatic System and Its Potential Pharmacological Modulation in Neurodegenerative Diseases. J. Clin. Med..

[189] Salman M.M., Kitchen P.J., Halsey A.M., Wang M.E., Törnroth-Horsefield S., Conner A.C., Badaut J., Iliff J.J., Bill R.M. (2021). Emerging Roles for Dynamic Aquaporin-4 Subcellular Relocalization in CNS Water Homeostasis. Brain J. Neurol..

[190] Szu J.I., Binder D.K. (2022). Mechanisms Underlying Aquaporin-4 Subcellular Mislocalization in Epilepsy. Front. Cell. Neurosci..

[191] Rong H., Jin L., Wei W., Wang X., Xi Z. (2015). Alpha-Synuclein Is a Potential Biomarker in the Serum and CSF of Patients with Intractable Epilepsy. Seizure.

[192] Paudel Y.N., Angelopoulou E., Piperi C., Othman I., Shaikh M.F. (2020). Revisiting the Impact of Neurodegenerative Proteins in Epilepsy: Focus on Alpha-Synuclein, Beta-Amyloid, and Tau. Biology.

[193] Mohammed S.R., Elmasry K., El-Gamal R., El-Shahat M.A., Sherif R. (2023). Alteration of Aquaporins 1 and 4 Immunohistochemical and Gene Expression in the Cerebellum of Diabetic Albino Rat. Tissue Cell.

[194] Lv Y.Q., Yuan L., Sun Y., Dou H.-W., Su J.-H., Hou Z.-P., Jia Yi Li, Li, W (2022). Long-Term Hyperglycemia Aggravates α-Synuclein Aggregation and Dopaminergic Neuronal Loss in a Parkinson’s Disease Mouse Model. Transl. Neurodegener..

